# Concurrent hypermethylation of DNMT1, MGMT and EGFR genes in progression of gliomas

**DOI:** 10.1186/1746-1596-7-8

**Published:** 2012-01-20

**Authors:** Éva Gömöri, József Pál, Bernadett Kovács, Tamás Dóczi

**Affiliations:** 1 Department of Pathology, Faculty of Medicine, Pécs University, Hungary; 2 Department of Neurosurgery, Faculty of Medicine, Pécs University, Hungary; 3 Clinical Neuroscience Group of the Hungarian Academy of Science, Faculty of Medicine, Pécs University, Hungary

## Abstract

**Background:**

Gliomas are the most common neoplasm of the brain. High-grade gliomas often resist treatment even with aggressive surgical resection and adjuvant radiation and chemotherapy. Despite the combined treatment, they frequently recur with the same or higher-grade histology. Genetic instability is commonly associated with inactivation of the normal DNA repair function and tumour suppressor genes as well as activation of oncogenes resulting from alterations of promoter hypermethylation, but the molecular mechanisms of the histological and clinical progression of gliomas are still poorly understood.

**Methods:**

This study involved longitudinal analysis samples of primary and recurrent gliomas to determine whether the progression of low- and high-grade gliomas is associated with the promoter methylation of the DNMT1, MGMT and EGFR genes by PCR-based restriction enzyme assay. Epigenetic inactivation of these three important glioma-associated genes was analyzed in paired biopsy samples from 18 patients with tumour recurrence.

**Results:**

The methylation analysis of the CpG sites in the DNA methyltransferase (DNMT1) promoter revealed a total of 6 hypermethylations (6/18), the methylguanine-DNA methyltransferase (MGMT) promoter revealed a total of 10 hypermethylations (10/18) and the epithelial grow factor receptor (EGFR) promoter revealed a total of 12 (12/18) hypermethylations respectively in recurrent gliomas. The results demonstrated that DNMT1 promoter hypermethylation does not occur in low-grade gliomas, it was mainly observed in secondary glioblastomas. Additionally, the MGMT and EGFR promoter was hypermethylated in both low-and high-grade GLs and their corresponding histological transformed GLs.

**Conclusion:**

This study has provided further evidence that the histological transformation and progression of gliomas may be associated with the inactivation of the EGFR and MGMT genes. It seems that EGFR and MGMT promoter hypermethylations are early events in the clonal evolution of gliomas and this gene inactivation has proved to be stable even in tumour recurrence. However, the DNMT hypermethylation is a late part of glioma progression.

**Virtual slides:**

The virtual slide(s) for this article can be found here: http://www.diagnosticpathology.diagnomx.eu/vs/1935054011612460

## Background

High-grade gliomas (GLs) often resist such treatment as complete surgical resection and chemotherapy in combination with radiation therapy. The lesions frequently recur after an asymptomatic period [1]. Most malignant gliomas are glioblastomas (GB) with clinical, histological, genetic and prognostic heterogeneities. Depending on their clinical and genetic characteristics, GBs have been divided into primary and secondary subtypes [2]. Primary GBs may develop rapidly without clinical or histological evidence of a precursor lesion with a low grade of malignancy. Secondary GB is the final stage of progression of a low-grade or anaplastic astrocytoma. Several lines of evidence indicate that multiple genetic abnormalities are associated with the development of GBs, such as the inactivation or amplification of several genes [3,4], the loss of heterozygosity of different chromosomes and microsatellite instability [5,6] with different mRNA and protein expression profiles [7]. The epidermal growth factor receptor (EGFR) proto-oncogene is a member of the HER/ERB-B family of transmembrane tyrosine receptor kinases. The overexpression of EGFR is responsible for cell proliferation, tumour cell migration and progression, as well as for the prognosis and the survival [8]. The complex genetic characterization indicates that primary GB is predominantly associated with the overexpression and amplification of EGFR with unfavourable biological behaviour. However, the EGFR amplification is less specific in secondary GB, though it has been shown that EGFR overexpression is a relatively late event in the dedifferentiation of glia-tumor cells. The epigenetic regulation and transcriptional inactivation of EGFR by promoter hypermethylation may play role in an absence of EGFR gene expression during GL progression. Furthermore, the hypermethylation of EGFR might be a possible explanation of the silence of this gene in secondary GB. Although a large numbers of epigenetic data have accumulated but no follow-up studies have been reported in which the promoter methylation of EGFR genes in tumour cells of primary and secondary GB were compared before and after the clinical recurrence and histological progression. The characterization of the EGFR methylation status of GB seems to be a critical issue in the delineation of the prognosis of these tumours.

Recent epigenetic studies have evidence that the promoter hypermethylation of CpG islands can be regarded as a common mechanism of inactivation of tumour-related genes [9]. The methylation of genomic DNA is performed by DNA methyltransferase (DNMT) which transfers the methyl groups to cytosine residues during DNA replication [10]. Aberrant DNA methylation has been reported in human cancer [11-13] with the involvement of the hypermethylation of tumour suppressor genes and the hypomethylation of oncogenes [14]. No studies on the DNMT methylation status of recurrent GLs are available.

Methylguanine-DNA methyltransferase (MGMT) is a DNA repair protein that directly and specifically removes mutagen DNA precursor and consequently causes resistance to alkylating drugs. The intracellular level of MGMT varies among tumours of the same histological type. Approximately one-third of GLs lack MGMT [15]. The MGMT gene does not commonly undergo mutation or deletion. A reduced MGMT expression may be caused by epigenetic inactivation. Promoter hypermethylation of MGMT is frequent in the process leading to the development of secondary GBs [16] and oligodendrogliomas [17].

To determine whether the progression of high-grade GLs is associated with the promoter methylation of the EGFR, DNMT1 and MGMT genes, we performed methylation analysis in paired samples of primary and recurrent GLs with or without histological progression. The results indicated that the hypermethylation of the EGFR and MGMT genes occurs in both primary and recurrent high-grade GLs and that the methylation profile was stable during GL progression. The promoter hypermethylation of the EGFR and MGMT genes suggests an important epigenetic regulation of GL progression. DNMT1 hypermethylation was not found in low-grade GLs, it was associated with secondary GBs.

## Materials and methods

### Pathological samples

Sequential brain tumour biopsy samples from 18 patients with GL were selected for the molecular analyses. All tumour specimens were cut into two; one half of the sample was paraffin-embedded for histological diagnosis of the tumours, while the other half were frozen for molecular analysis. The histological findings on the two halves were identical. Neither the high-grade nor the low-grade GLs displayed morphological heterogeneity. During the selection of the slides, the necrotic areas were omitted. Tissues derived from paraffin-embedded material and frozen samples were sufficient for the histological diagnosis and DNA preparation (all DNA preparations with a high standard DNA concentration).

**Figure 1:**
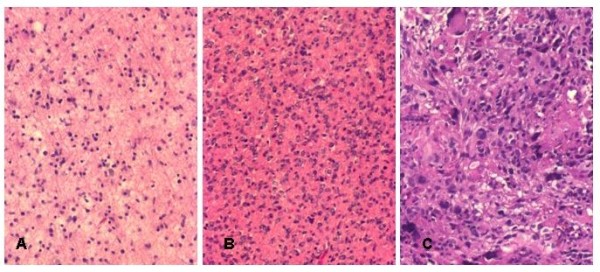
**Photomicrographs show the different histological type of recurrent GL's**. Moderately cellular tumour composed of uniform astrocytic cells concerning diffuse astrocytoma, Grade II (A). Histological features of anaplastic astrocytoma with hypercellularity and hyperchromatic, irregular nuclei, Grade III (B). Glioblastoma with high grade anaplasia and giant cells, Grade IV (C).

Diagnoses were established by means of the WHO classification [18]. The histological findings on the first biopsy were classified into three GL categories: diffuse astrocytomas (A2) (cases 1-6); anaplastic astrocytoma (A3) (cases 7-10) and glioblastoma (GB) (cases 11-18) (Figure 1). In 8 patients, the histological picture of the second biopsy sample was identical with that of the primary tumour. In 10 patients, the histological finding on the primary tumour was transformed into the higher-grade at the second biopsy. Sixteen patients received postoperative radiation therapy, 10 patients underwent postoperative chemotherapy such as a Temodal and Carbo/VP-16 combination and only 1 patient did not participate in postoperative therapy (Table 1).

**Table 1:** Summary of the clinical and histological data of 18 patients with primary and recurrent gliomas

Case	Age (y)	Sex	Site of tumor	Interval of biopsies (m)	Survival (m)	Histology P/R	Adjuvant therapy
1	20	M	RTL	15	ALIVE	PA2	R
			RTL			RA3	-

2	48	M	LTL	24	ALIVE	PA2	R
			LTL			RA3	-

3	55	M	RFL	13	14	PA3	-
			RFL			RA3	-

4	50	F	LTL	74	ALIVE	PA2	R
			LTL			RA3	T

5	30	M	RFL	662	ALIVE	PA2	R
			RFL			RA3	-

6	41	F	RTL	42	64	PA2	R
			RTL			RA3	-

7	50	M	LOL	9	18	PA3	R
			LOL			RGB	T

8	15	F	LFL	31	49	PA3	R
			LFL			RGB	CVP

9	27	F	RFL	33	98	PA3	R
			RFL			RGB	T

10	56	F	RPL	12	32	PA3	R
			RPL			RGB	-

11	69	F	RFL	11	16	PGB	R
			RFL			RGB	-

12	44	M	LPL	3	6	PGB	R
			LPL			RGB	-

13	47	M	LFL	9	12	PGB	-
			LFL			RGB	T

14	55	M	LTL	13	19	PGB	R
			LTL			RGB	T

15	62	M	RFL	6	18	PGB	R
			RFL			RGB	T

16	54	M	RTL	12	14	PGB	R
			RTL			RGB	T

17	41	M	RTL	10	24	PGCGB	R
			RTL			RGCGB	T

18	17	F	RTL	15	29	PGCGB	R
			RTL			RGCGB	CVP

Abbreviations: Histology: P for primary and R for recurrence; A2, diffuse astrocytoma; A3, anaplastic astrocytoma; GB, glioblastoma; GCGB, giant cell glioblastoma; LFL, left frontal lobe; RFL, right frontal lobe; LTL, left temporal lobe; RTL, right temporal lobe; RPL, right parietal lobe; ROL, right occipital lobe; R, radiotherapy; CVP, Carbo/VP-16 chemotherapy; T, Temodal; female; M, male; y, year; m, month; Interval is the time in months between the two biopsies

### DNA extraction

In each case, genomic DNA was extracted from formaldehyde-fixed and paraffin-embedded tissue samples and cryopreserved tumour samples, using DNA extraction kits (paraffin-embedded tissue with the High Pure PCR Template Preparation Kit from Roche Diagnostics, USA, and frozen tissue with the NucleoSpin Tissue Kit from Manchery-Nagel, Germany) according to the manufacturer's instructions.

### Analysis of EGFR, DNMT1 and MGMT methylation

Methylation-specific PCR [19] was used to analyze genomic DNA samples for the presence of hypermethylation at the CpG sites of the EGFR, DNMT1 and MGMT genes. The gene sequence contains HpaII and MspI restriction sites. DNAs were digested with HpaII (Promega Corporation, Madison, WI, USA) and MspI (Promega Corporation, Madison, WI, USA) restriction endonucleases in a volume of 20 μl, according to the manufacturer's instructions. Digested and undigested DNAs were PCR-amplified by using primer pairs for EGFR exon 18: 5'- AGGCGTACATTTGTCCTTCC-3' (forward) and 5'-TGGAGTTTCCCAAACACTCAG-3' (reverse), DNMT1 exon 23: 5'-GCAGATGCACTGTGGAGAAG-3' (forward) and 5'- CTCTTCTCAGGGGCAAACAG-3'(reverse), MGMT exon 5: 5'- ACAGGTGTTTGCCCGTTTAG-3' (forward) and 5-AAGTGTTGGAGTGGGTGGAG-3'(reverse).

PCR was performed with a Perkin-Elmer Model 2700 PCR system (Foster City, CA, USA) with the following PCR program: initial denaturation at 94 oC for 5 min, followed by 30 cycles of denaturation at 94 oC for 45 s, annealing at 60 oC for 45 s, extension at 72 oC for 45 s, and a final elongation step at 72 oC for 5 min. Electrophoretic separation was achieved in 2% agarose gels, with staining with ethidium bromide, and visualization under UV illumination. Unmethylated and methylated controls were included in all reactions.

### Immunohistochemical reaction of EGFR, DNMT1 and MGMT

After microwave antigen retrieval procedure the sections were incubated with the anti-EGFR monoclonal antibody (DAKO Glostrup, Denmark, RTU), anti- DNMT1 monoclonal antibody (BIOZOL, Germany 1:500) and anti-MGMT monoclonal antibody (Novobius, USA 1:50). Binding was visualized with biotinylated IgG using the avidin-biotin peroxidase detection system (Vectastain ABC Universal Elite Kit, Vector) and 3,30-diamino-benzidine (DAB) as the chromogenic substrates. Control sections were treated similarly except the primary antibody was omitted from the procedure. The quality of immunostaining slightly varied due to the degree of fixation which differed in each case.

## Results

### Analysis of DNMT1 promoter methylation in primary and recurrent GLs

To evaluate the methylation of CpG sites in the DNMT1, EGFR and MGMT promoters, PCR products amplified from undigested, and from HpaII and MshI-digested DNAs were evaluated in parallel. In each case, the results of DNMT1, EGFR and MGMT promoter methylation of the first and second biopsy samples were compared (Table 2).

**Table 2:** Summary of methyaltion conditions and immunoreactivity of 18 patients with primary and recurrent gliomas

Cases	HT	DNMT		EGFR		MGMT	
		PGL	RGL	PGL	RGL	PGL	RGL
1	A2 → A3						

2	A2 → A3			-	-		

3	A3 → A3					-	-

4	A2 → A3			-	-	-	-

5	A2 → A3				+	-	-

6	A2 → A3						

7	A3 → GB	-	-	-	-	-	-

8	A3 → GB	-	-	-	-	-	-

9	A3 → GB		-	-	-		

10	A3 → GB	-	-	-	-	-	-

11	GB → GB	-	-	-	-		

12	GB → GB	-	-	-	-	-	-

13	GB → GB			-	-		-

14	GB → GB			-	-	-	-

15	GB → GB			-	-		

16	GB → GB					-	-

17	GCGB → GCGB						

18	GCGB → GCGB						

Abbreviations: no mark, hypermethylation; oval mark, unmethylation; PGL, primary glioma; RGL, recurrent glioma;HT, histological tranformation; A2, diffuse astrocytoma; A3, anaplastic astrocytoma; GB, glioblastoma; GCGB, giant cell glioblastoma; + positive immunoreactivity; - negative immunoreactivity

**Figure 2:**
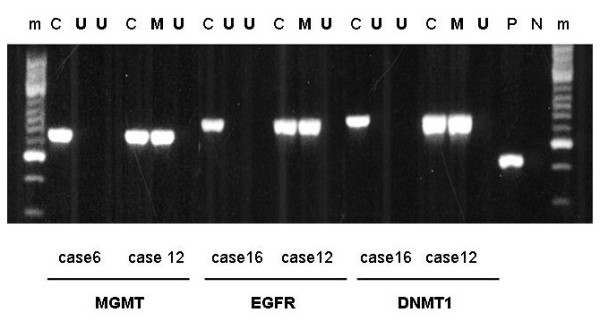
**Representative methylation analysis of CpG islands in the MGMT, EGFR and DNMT1 promoter in GL samples of cases 6, 12 and 16**. C, undigested DNA samples; P, β2- microglobulin as a positive control; N, no DNA sample as a negative control; U, unmethylated DNA samples; M, methylated DNA samples, m, molecular weight marker

DNMT1 promoter methylation was present in 6 of the 18 cases (33.3%) in 5 primary and 6 recurrent GLs. In 5 samples of GLs and the corresponding recurrent samples (cases 7, 8, 10, 11 and 12) and in one only recurrent sample (case 9), the DNMT1 promoter region was resistant to digestion by HpaII and sensitive to digestion by MspI. The representative cases are illustrated in Figure 2. At the first biopsy all of the HpaII-resistant cases were high-grade GLs, involving either anaplastic astrocytoma or GB. At the second biopsy, all of the HpaII-resistant cases were GBs. In cases 7, 8 and 10, the primary biopsies were anaplastic astrocytomas that transformed to GBs as in case 9. In cases 11 and 12, both biopsies were GBs that showed no histological alteration. All those low-grade GLs including a diffuse astrocytoma (case 1-6), at the first biopsy, that showed histological progression transform to anaplastic GLs. The DNMT1 promoter region was sensitive to digestion by both HpaII and MspI in only 1 anaplastic astrocytoma (case 9) and 6 primary GBs that exhibited no histological alteration (cases 13-18),

### Analysis of EGFR promoter methylation in primary and recurrent GLs

EGFR promoter methylation was present in 12 of the 18 cases (66.6%) in 11 primary and 12 recurrent GLs. In 11 samples of GLs and the corresponding recurrent samples (cases 2, 4, and 7-15) and in one second sample (case 5), the EGFR promoter region was resistant to digestion by HpaII and sensitive to digestion by MspI (Figure 2). As concerns the first biopsy samples, the HpaII-resistant cases were either low- or high-grade GLs; at the second biopsy, including anaplastic GL or GB all of the HpaII-resistant cases were high-grade tumour. Low-grade GLs, the diffuse astrocytomas (cases 2 and 4), and their corresponding high-grade tumours were HpaII-resistant. Four primary biopsy cases categorized as anaplastic astrocytoma (7-10) that transformed into GBs and both samples of 5 primary GBs (cases 11-15) were also HpaII-resistant. Only one anaplastic astrocytoma was HpaII-resistant (case 5). The other low-grade GLs, diffuse astrocytoma (cases 1, 3, 5 and 6) that showed histological progression to anaplastic GLs, 1 primary GB (case 16) and 2 giant cell GB (cases 17 and 18) the EGFR promoter region was sensitive to digestion by both HpaII and MspI.

### Analysis of MGMT promoter methylation in primary and recurrent GLs

MGMT promoter methylation was present in 10 of the 18 cases (55.5%), in 9 primary and 10 recurrent GLs. In 9 samples of GLs and the corresponding recurrent samples (cases 3-5, 7, 8, 10, 12, 14 and 16) and in 1 recurrent sample (case 13), the MGMT promoter region was resistant to digestion by HpaII and sensitive to digestion by MspI (Figure 2). At the first biopsy, the HpaII-resistant cases proved to be both low- and high-grade GLs. All of the HpaII-resistant cases at the second biopsy were high-grade tumours including anaplastic GL or GB. Low-grade diffuse astrocytomas (cases 3, 4, 5) were HpaII-resistant. Three anaplastic astrocytomas (cases 7, 8 and 10) that transformed into GBs were resistant to digestion by HpaII. In 3 primary GBs (cases 12, 14 and 16), both biopsies were HpaII-resistant; in case 13, only second biopsy was HpaII-resistant. The MGMT promoter region of the progressive low-grade astrocytoma (cases 1, 2 and 6), 1 secondary GB (case 9) and 5 primary GBs (cases 11, 13, 15, 17 and 18) were sensitive to digestion by both HpaII and MspI.

### DNMT1, EGFR and MGMT immunolabeling in recurrent GLs

Immunohistochemical reaction confirmed results of methylation analysis. DNMT1-immunoreactivity was present at all unmethylated samples (case 1-6 and case 13-18 and first biopsy of case 9). However, all methylated tumours showed negative immunohistochemistry (case 7-12 except first biopsy of case 9).

EGFR immunohistochemical reactions revealed no labelling in methylated samples (cases 2, 4, and 7-15) and both biopsies of case 16. The other tumour's pairs showed membrane immunpositivity (cases 1, 3, 5, 6, 17 and 18).

**Figure 3:**
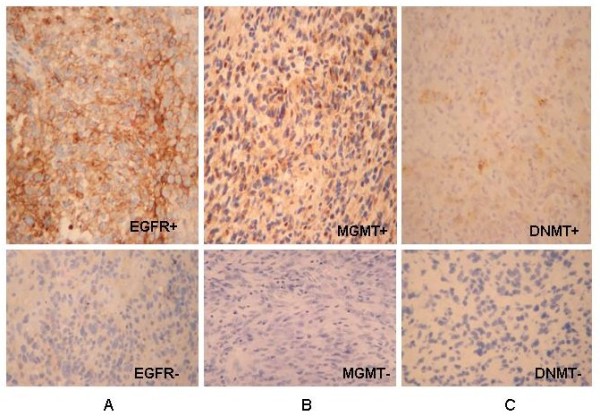
**Photomicrographs show immunohistochemical reactions of EGFR, MGMT and DNMT antibodies**. Recurrent anaplastic astrocytoma (case 6) was strong EGFR membrane positive but no reaction in glioblastoma (case 10) (A). MGMT immunostaining of nuclei with a weak cytoplasmic staining in primary glioblatoma (case 11) but no reaction in anaplastic astrocytoma (case 7) (B). Recurrent anaplastic astrocytoma (case 4) was DNMT cytoplasmic positive but no reaction in seconder glioblastoma (case 8) (C).

MGMT nuclear immunoreactivity was present at all unmethylated samples (cases 1, 2, 6, 9, 11, 15, 17 and 18). However, all methylated tumours showed negative immunohistochemistry (case 3-5, 7, 8, 10, 12-14 and 16). The representative results are illustrated in Figure 3.

## Discussion

Genetic instability can be generated by the epigenetic regulation of multiple genes, including oncogenes, tumour suppressor genes and mismatch repair genes [20]. The methylation mechanism plays an important role in the pathogenesis of the recurrence of GLs [21]. In order to establish whether the histological transformation and clinical progression of GLs are associated with the epigenetic inactivation of three important GL-associated genes, paired biopsy samples were analyzed for the functionally important methylated exons of the DNMT1, MGMT and EGFR genes to elucidate their methylation patterns. No DNMT promoter methylations were found in the low-grade GLs. Promoter methylations of DNMT1 were not characteristic (2/8) in primary GBs; all secondary GBs were hypermethylated (4/4). This is the first presentation that the low-grade GLs are not and primary GBs are less associated with DNMT1 gene hypermethylation. These findings suggest that the methylation mediated normal transcriptional regulation of DNMT1 gene has being worked in the early stage of GLs' proliferation. Additionally, the DNMT1 gene inactivation by promoter methylation develops in a later stage of GLs, in secondary GB. We have assumed that the DNMT1 gene is involved in normal DNA methylation in low grade gliomas. However, the epigenetic inactivation of DNMT1 gene plays a part in the pathogenesis of anaplastic astrocytoma progression.

The MGMT promoter was hypermethylated in both low- and high-grade GLs. Furthermore, the hypermethylation accumulated during histological progression of the GLs. Our results demonstrate that the development of both primary and recurrent GLs is associated with MGMT gene hypermethylation and it is more frequent in high-grade GLs [16]. The distribution of MGMT promoter hypermethylation shows that it starts to develop at an early stage of GL and the gene inactivation is stable even on tumour progression. The promoter methylation of the MGMT gene compromises DNA repair and has been associated with a longer progression-free survival in GLs' patients [22]. Further, recent studies indicate that MGMT promoter methylation has strong prognostic relevance even in anaplastic gliomas but irrespective of sensitivity of alkiling agents [23].

Several lines of evidence suggest that the hypermethylation of oncogenes and tumour suppressor gene participate in the development of GLs pointing to a wide-ranging increase in methylation activity in glial tumour genesis [24,25]. This is supported by the findings that EGFR promoter hypermethylation occurs in solid tumour cell lines, e.g. breast, colon and lung cancers [26]. The EGFR promoter was hypermethylated in both low- and high-grade GLs and their corresponding histological transformed GLs, but the number of alterations was not increased considerably in progression. Our results also indicate that the development of both type of GBs are associated with EGFR gene hypermethylation. These data suggest that *de novo *GBs are regulated by epigenetic inactivation of EGFR gene besides the well-known EGFR gene amplification and overexpression (2). On the other hand, the hypermethylation of the EGFR promoter is typical event *of secondary *GBs. Additionally, tumours with EGFR hypermethylation are more resistant to tyrosine kinase inhibitor, which indicates a poorer prognosis [26,27]. The current evidence indicates that gene transcriptional regulation begins in the early stage of tumour genesis and the change in DNA methylation is a principal component of tumour progression [28,29]; but it unlikely to be involved in effectivity of chemotherapy [23].

The simultaneous evaluation of methylation analysis and immunohistochemical reaction revealed similar results, suggesting that CpG methylation mechanism regulates gene activation during glioma progression. It seems that the methylation mediated inactivity of genes associated with loss of protein expression in recurrent GL's. However, the inverse relation between methylation status and immunoreactivity in case 5, 13 and 16 is probably caused by genetic abnormalities including overexpression or deletion of EGFR and MGMT genes.

In conclusion, this study has provided epigenetic evidence that promoter hypermethylation of all three important GL-associated genes, DNMT1, MGMT and EGFR may play a role in GL progression and the inactivation of these genes is stable even as concerns tumour recurrence. EGFR and MGMT promoter hypermethylation was revealed in low-grade GLs and their anaplastic counterparts. This study has provided an indication that the histological transformation and recurrence of GLs may be associated with EGFR and MGMT promoter hypermethylation, which are early events in the development or clonal evolution of GLs. Additionally, DNMT1 gene is still working entirely in the early GLs' proliferation, the hypermethylated inactivation associated mainly with late phase of GLs' progression.

## Abbreviations

DNA: Deoxyribonucleic acid; DNMT: DNA methyltransferase; MGMT: Methylguanine-DNA methyltransferase; EGFR: Epithelial growth factor receptor; PCR: Polymerase Chain Reaction; GL: glioma; GB: lioblastoma; GCGB: Giant cell glioblastoma; WHO: World Health Organization; A2: Diffuse astrocytoma; A3: Anaplastic astrocytoma; CVP, Carbo/VP-16: Carboplatin and etoposide chemotherapy; UV: Ultraviolet; P: Primary tumour; R: Recurrent tumour; LFL: Left frontal lobe; RFL: Right frontal lobe; LTL: Left temporal lobe; RTL: Right temporal lobe; RPL: Right parietal lobe; ROL: Right occipital lobe; R: Radiotherapy; T: Temodal; F: Female; M: Male; Y: year; M: month; I: Interval is the time in months between the two biopsies; H: Histological transformation; PGL: Primary glioma; RGL: Recurrent glioma; RTU: Ready to use.

## Competing interests

The authors declare that they have no competing interests.

## Authors' contributions

JP carried out the analysis of genes promoter methylation studies. BK performed the immunohistochemical reactions. EG conceived the study and participated in the publication of the manuscript. TD participated in its design and coordination. All authors read and approved the final manuscript.
